# Dosimetric Impact of Inter-Fraction Variability in the Treatment of Breast Cancer: Towards New Criteria to Evaluate the Appropriateness of Online Adaptive Radiotherapy

**DOI:** 10.3389/fonc.2022.838039

**Published:** 2022-04-11

**Authors:** Martina Iezzi, Davide Cusumano, Danila Piccari, Sebastiano Menna, Francesco Catucci, Andrea D’Aviero, Alessia Re, Carmela Di Dio, Flaviovincenzo Quaranta, Althea Boschetti, Marco Marras, Domenico Piro, Flavia Tomei, Claudio Votta, Vincenzo Valentini, Gian Carlo Mattiucci

**Affiliations:** ^1^ Università Cattolica del Sacro Cuore, Rome, Italy; ^2^ Dipartimento Diagnostica per Immagini, Radioterapia Oncologica ed Ematologia, Fondazione Policlinico Universitario Agostino Gemelli IRCCS, Rome, Italy; ^3^ UOC Radioterapia Oncologica, Mater Olbia Hospital, Olbia, Italy

**Keywords:** AI radiotherapy, predictive modeling, CBCT radiotherapy, inter-fraction dose variation, online adaptation

## Abstract

**Purpose:**

As a discipline in its infancy, online adaptive RT (ART) needs new ontologies and *ad hoc* criteria to evaluate the appropriateness of its use in clinical practice. In this experience, we propose a predictive model able to quantify the dosimetric impact due to daily inter-fraction variability in a standard RT breast treatment, to identify in advance the treatment fractions where patients might benefit from an online ART approach.

**Methods:**

The study was focused on right breast cancer patients treated using standard adjuvant RT on an artificial intelligence (AI)-based linear accelerator. Patients were treated with daily CBCT images and without online adaptation, prescribing 40.05 Gy in 15 fractions, with four IMRT tangential beams. ESTRO guidelines were followed for the delineation on planning CT (pCT) of organs at risk and targets. For each patient, all the CBCT images were rigidly aligned to pCT: CTV and PTV were manually re-contoured and the original treatment plan was recalculated. Various radiological parameters were measured on CBCT images, to quantify inter-fraction variability present in each RT fraction after the couch shifts compensation. The variation of these parameters was correlated with the variation of V95% of PTV (ΔV95%) using the Wilcoxon Mann–Whitney test. Fractions where ΔV95% > 2% were considered as adverse events. A logistic regression model was calculated considering the most significant parameter, and its performance was quantified with a receiver operating characteristic (ROC) curve.

**Results:**

A total of 75 fractions on 5 patients were analyzed. The body variation between daily CBCT and pCT along the beam axis with the highest MU was identified as the best predictor (*p* = 0.002). The predictive model showed an area under ROC curve of 0.86 (95% CI, 0.82–0.99) with a sensitivity of 85.7% and a specificity of 83.8% at the best threshold, which was equal to 3 mm.

**Conclusion:**

A novel strategy to identify treatment fractions that may benefit online ART was proposed. After image alignment, the measure of body difference between daily CBCT and pCT can be considered as an indirect estimator of V95% PTV variation: a difference larger than 3 mm will result in a V95% decrease larger than 2%. A larger number of observations is needed to confirm the results of this hypothesis-generating study.

## Introduction

In recent years, technological evolution and the advent of artificial intelligence (AI) have led to incredible improvements in the fight against cancer, opening treatment scenarios that were unthinkable just a few years ago (1).

In the field of radiation therapy (RT), the new cutting-edge technologies are able to modify online the RT treatment plan to effectively compensate for the patient anatomical variability, which is present during different treatment days, in a procedure known as online adaptive radiotherapy (ART) (2).

The modern technologies implementing online ART aim to integrate advanced AI-based systems to speed up the on-table adaptive procedure, to shorten the treatment slot time and avoid un-addressable organ variation that may occur during on-table ART procedure (3, 4).

To date, the RT systems licensed for online ART use the positioning images acquired through on-board MR scanners or Cone Beam Computed Tomography (CBCT) systems to elaborate the adapted treatment plans, with treatment slot times ranging from 15 to about 60 min, depending on the technology used and the case complexity (5–7).

Being a discipline in its infancy, there is an increasing need to define common ontologies and specific criteria to evaluate the appropriateness of online ART treatments. The benefits offered by such treatments are in fact a matter of study: although in some districts, such as the abdomen, the advantages offered by this approach are well demonstrated in the current literature, in others, the benefits are still under investigation, as they have to be balanced with the efforts required, in terms of both staff resources and patient stress (8–11).

Breast cancer is one of the anatomical sites in which the benefit of online ART is still under investigation: the adjuvant RT approach is in fact already very effective, as demonstrated by the results of several clinical trials present in literature with long-term outcome. In 2011, the Early Breast Cancer Trialists’ Collaborative Group (EBCTCG) reported the results of a meta-analysis of 10,801 women treated with radiotherapy after breast conservative surgery, demonstrating that the use of adjuvant RT significantly reduced the risk of any first (locoregional or distant) recurrence and breast cancer mortality (12). A similar evidence was observed in patients treated with RT after mastectomy (13).

Recent experiences report percentages of local and regional recurrence after breast-conserving surgery followed by RT ranging from 7% to 13%, often associated to initial tumor size (14, 15).

However, despite the fact that local recurrences can be considered not common events, it is plausible to suppose that online ART treatments could contribute to further reduce such evidence, mainly in selected cases where the inter-fraction variability may lead to compromise the target coverage with respect to the prescribed dose, thus increasing the risk of local recurrence.

The aim of this study is to propose new evaluation criteria to identify which patients affected by right-side breast cancer could benefit in receiving online ART treatment, considering an RT treatment administered in intensity modulated radiation therapy (IMRT) modality.

Considering that the dose constraints related to nearby organs at risk (OARs) are widely met in the IMRT treatment of right breast, the main focus of attention is on the planning target volume (PTV) coverage with respect to the 95% of the prescribed dose, which has to be maintained higher than 95% as recommended by international guidelines (16, 17). For this purpose, it is necessary to quantify the dosimetric variation in the tumor coverage due to the daily inter-fraction variability; once compensated, the couch shifts, determined by the alignment of daily positioning images. Once such variability is quantified, a predictive model was also elaborated to correlate imaging parameters, related to patient positioning, to dosimetric effects on target coverage, with the idea of providing a valid tool to clinicians to know in advance the dosimetric impact of an inter-fraction variability effect.

## Materials and Methods

### Patients and Treatment Characteristics

The present study has a retrospective nature and was based on the analysis of five patients affected by right breast tumor, with age higher than 18 years. Patients showed a diagnosis of Early Breast Cancer (EBC), were of legal age, and signed an informed consent for data collection and anonymized analyses.

An adjuvant RT treatment was administered to all of them, prescribing 40.05 Gy in 15 fractions (2.67 Gy/fraction) to the whole breast. A sequential boost consisting of 10 Gy in 5 fractions to the tumor bed was also prescribed in selected patients, according to the disease stage and clinical risk factors. Treatments were performed at Mater Olbia Hospital (Olbia, Italy) using Varian Ethos™ (Varian Medical System, Mountain View, California, US) between August and September 2021.

A simulation computed tomography (CT) image was acquired for all free breathing patients using the dedicated 16-slice CT scanner (GE RT discovery, GE Healthcare, Chicago, Illinois) available in our department, keeping a slice thickness of 2.5 mm.

During CT simulation, the patient breathing motion was studied using a 4DCT acquisition in ten phases, and patients showing negligible sternum variation (less than 1 mm) in all the 4DCT phases were selected. Such selection was performed to limit the impact of breathing motion and ensure that the body variation object of the present study would be related to patient positioning.

Average CT image was reconstructed and used for therapy volume contouring and treatment planning. The clinical target volume (CTV) was outlined according to the ESTRO consensus guidelines and defined as the entire right mammary gland, while PTV was calculated as an anisotropic margin from CTV: 5 mm in the medio-lateral direction, 7 mm in the antero-posterior and cranio-caudal direction, with a crop margin of 5 mm from the body (18).

In case of the presence of prothesis, this was included in the CTV definition. Heart, esophagus, ipsilateral glenohumeral joint, spinal canal, spinal cord, thyroid gland, lungs, and contralateral breast were delineated and considered as OARs, subjected to the dose constraints reported in clinical experiences focused on breast cancer (19–22).

All the patients were treated following an intensity modulated RT (IMRT) technique consisting of four tangential beams, normalizing the treatment plan to the median target dose as recommended by ICRU Report 62 and 83 (16, 17). Treatments were administered without online adaptation.

During treatment therapy, all the CBCT acquisitions were performed using the longest acquisition time available for the thorax (30.8 s), to further reduce the impact of breathing motion on body variation.

For each patient, all the CBCT images acquired for patient positioning were rigidly aligned with the planning CT, excluding rotational shifts according to Ethos™ clinical workflow (23). Synthetic CT images were created transferring the Hounsfield Units (HU) from simulation CT to CBCT through a deformable registration.

The targets (CTV and PTV) and the nearby OARs (heart and lungs) were manually re-contoured on the daily images and treatment plan was recalculated considering the fluence of the original plan using Eclipse™ (Varian Medical System, Palo Alto, California) as treatment planning system (TPS), and Acuros™ XB (Varian Medical System, Palo Alto, California) version 15.6 as dose calculation algorithm (24).

### Definition of Criteria for Adaptive Appropriateness

For each treatment fraction recalculated, the values of V95% and V105% of PTV were registered and considered as target metric values. The deviations of V95% and V105% parameters of PTV with respect to the values reported in the original plan were registered for each treatment fraction.

On the basis of the deviations observed and the value of the dose constraints, the treatment fractions were categorized as follows:

✓ *Optimal* when the treatment fraction showed a dose deviation from the original plan in V95%(PTV) < 2% and the dose objectives V95% (PTV) >95% and V105% (PTV) <5% remain preserved.
✓ *Not Optimal* when the treatment fraction showed a dose deviation from the original in V95% (PTV) ≥ 2%. In particular, a *not optimal* fraction can be considered:
  ○ *Acceptable* if the dose objectives V95% (PTV) ≥95% and V105% (PTV) <5% remain preserved
  ○ *Unacceptable* (to be adapted) in case the treatment fraction does not ensure the V95% (PTV) ≥95% and/or the V105% (PTV) results to be higher than 5%

The appropriateness of moving towards an adaptive approach was then evaluated based on the numbers of fractions categorized as “*not optimal*” observed during the standard treatment.

### Predictive Model

Once the treatment fractions were classified into two categories, a predictive model was elaborated to quantify the probability of a treatment fraction categorized as *not optimal*, so that it could benefit from online adaptation.

**Figure 1:**
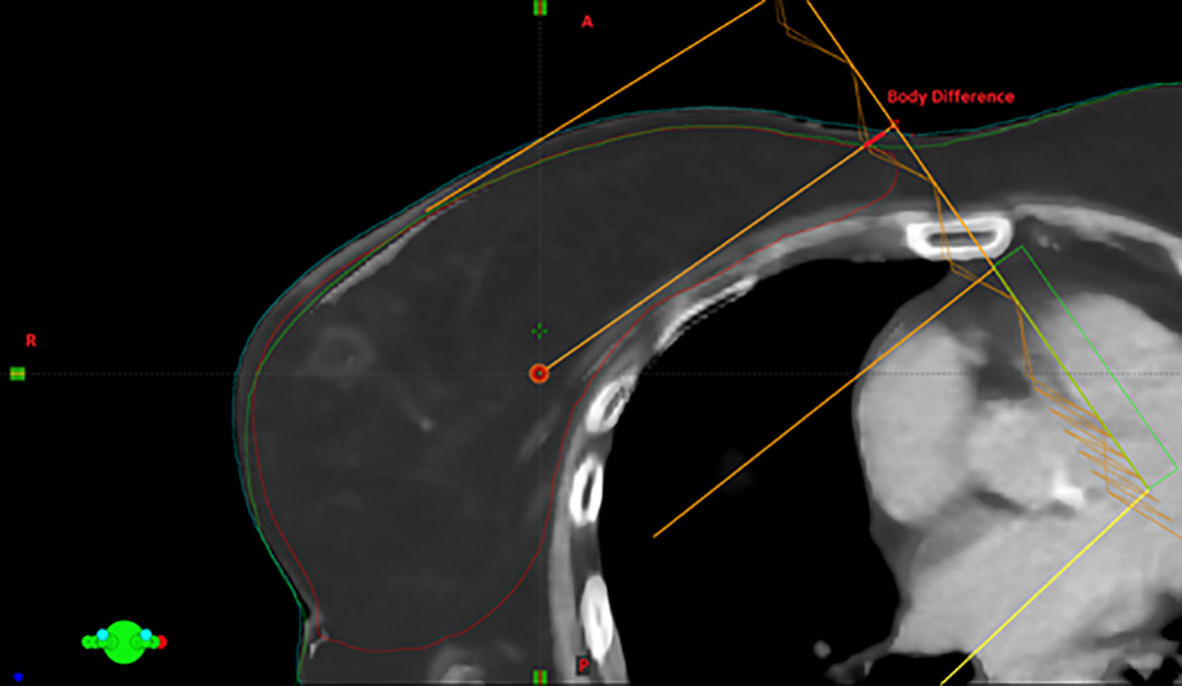
Visual example of the body variation measurement: the difference between the body in CBCT and the corresponding one in simulation CT along the beam axis with higher MUs is highlighted in red.

Various radiological parameters were measured on CBCT images, with the aim of quantifying inter-fraction variability present in each RT fraction after the couch shifts compensation. The absolute difference in terms of body between daily CBCT and pCT was calculated along each beam axis, considering the isocenter plan as the reference plan. An example of the radiological parameters measured is reported in **Figure 1**.

The absolute body difference was also calculated considering the whole PTV as cranio-caudal (CC) extension and the maximum values observed were considered as additional parameters.

The variation of these parameters was correlated with the *not acceptable* fractions using the Wilcoxon Mann–Whitney test or the *t*-test, depending on the distribution of the variable with respect to the outcome, which was preliminarily evaluated using the Shapiro–Wilk test (25). The Benjamini–Hochberg method was adopted to correct for multiple comparisons (26, 27).

Not optimal fractions were considered as adverse events. A logistic regression model was calculated considering the radiological parameter showing higher significance at the univariate analysis, and its performance was quantified in terms of receiver operating characteristic (ROC) curve (28).

The area under the ROC curve (AUC) was used as target metric, and the 95% confidence interval (95% CI) was defined by means of a bootstrap approach with 2,000 iterations. The best cutoff threshold was determined maximizing the Youden Index, and the values of sensitivity and specificity at the best threshold were calculated accordingly, as reported in similar experiences (28–30).

## Results

The clinical characteristics of the patients included in the study and the corresponding dosimetric values of the considered treatment plans are reported in **Table 1**. All the patients had negative margins after breast surgery and they were characterized by a molecular profile “luminal A”, which generally corresponds to low-grade tumors and a favorable prognosis (31). At the histological examination, all the patients received a diagnosis of ductal invasive carcinoma (non-special type).

**Table 1:** Clinical and dosimetric characteristics of the patients analyzed.

		Patient 1	Patient 2	Patient 3	Patient 4	Patient 5
**Clinical Characteristics**	**Age**	53	51	63	58	69
	**Grading**	G1	G2	G2	G1	G1
	**TNM Classification**	pT1b pN1a	pT1b pN0	pT2m pN0	pT1b pN1mi	pT1b pN0
	**Staging**	IIA	IA	IIA	IB	IA
**Dosimetric Characteristics**	**Beam 1 (MU)**	409.7	364.2	253.7	405.3	317.8
	**Beam 2 (MU)**	224.8	240.9	307.6	323.5	290.5
	**Beam 3 (MU)**	251.1	190	231.1	254.7	296.2
	**Beam 4 (MU)**	248.3	176.7	251	194.9	319.6
	**CTV volume (cc)**	955.8	577	589.7	835.8	1225.8
	**V95% PTV (%)**	98.2	98.8	99.8	98	98.4
	**V105% PTV (%)**	0	0	0	0	0
	**V20Gy Lung IPSI (%)**	15.6	12.1	157	13.2	13.3
	**Mean Dose Heart (Gy)**	0.97	0.7	1.82	0.94	1.04
	**Max Dose Spinal Canal (Gy)**	0.54	0.52	0.53	0.7	0.69

**Figure 2:**
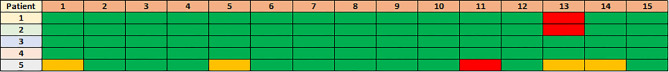
Visual representation of the treatment fractions analyzed for each patient. The optimal fractions are in green, the non-optimal but acceptable fractions are in yellow, and the fractions requiring online adaptation are in red.

A total of 75 fractions on 5 patients were analyzed: a general overview of the analysis of the treatment fractions is reported in **Figure 2**.

Out of a total of 75 fractions, 7 were found to be *not optimal*: specifically, three were not acceptable and four were acceptable. All the cases investigated showed the V105% of PTV always lower than 5%, so cases classified as *not optimal* are due to deviation related to V95% of PTV.

A patient with a larger initial CTV (Patient 5) was the case reporting the higher number of fractions that required online adaptation (5). As regards the results observed at the univariate analysis, the body variation along the beam axis with the highest MU was identified as the best predictor (*p* = 0.002).

**Figure 3:**
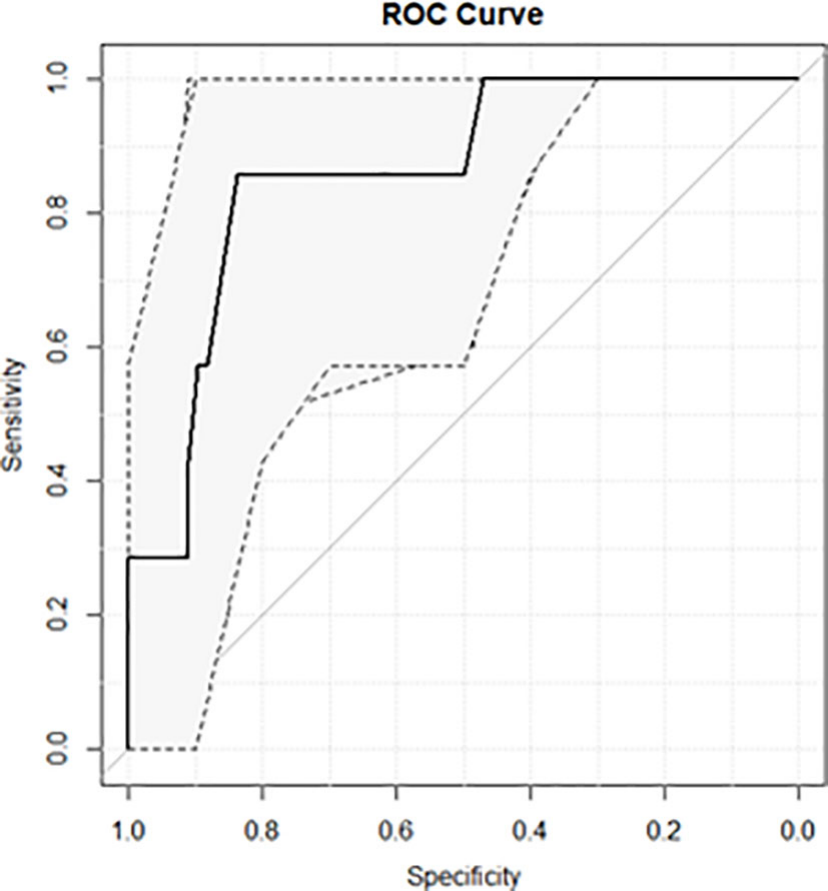
ROC curve of the predictive model focused on identifying treatment fractions where a variation higher than −2% was observed in V95% of PTV.

The ROC curve of the model elaborated using such parameter is reported in **Figure 3**: it shows an AUC of 0.86 (95% CI, 0.82–0.99) with a sensitivity of 85.7% and a specificity of 83.8% at the best cutoff threshold, which was identified to be equal to 3 mm and to correspond to a Youden Index of 0.69.

**Figure 4:**
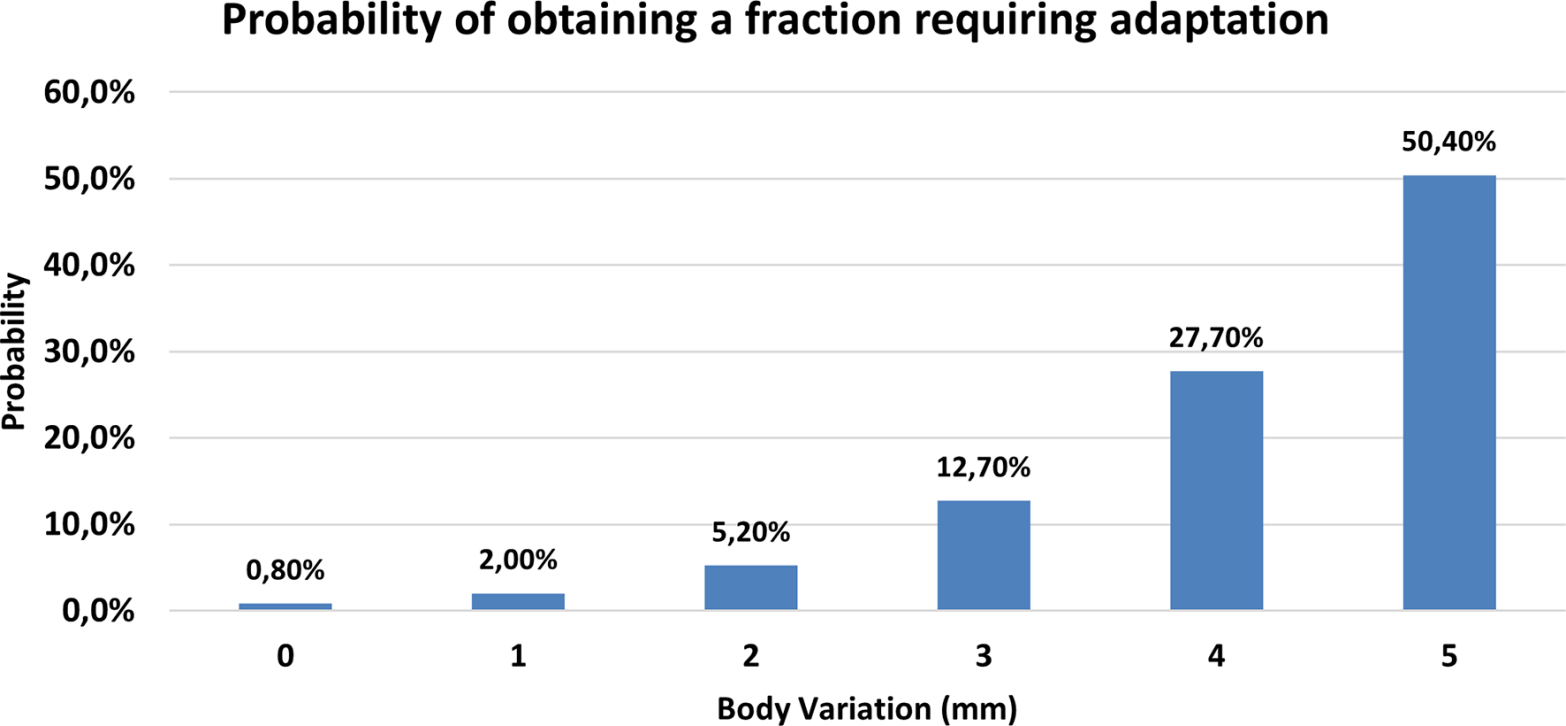
Probability of obtaining a fraction requiring adaptation as a function of body variation measured along the beam axis with the highest MU.

**Figure 4** reports the probability of obtaining a treatment fraction requiring online adaptation as a function of the body variation along the beam axis with the highest MU.

## Discussion

Patient selection is becoming a fundamental topic in the context of online ART treatments, and new criteria to identify patients who may effectively benefit from these technologies are needed (10, 32). 

Compared to conventional RT, the online ART is in fact more time-consuming and requires a very experienced and committed staff, so its use has to be focused on selected cases (33–35).

It is reasonable to assume that such selection criteria would be disease-specific and also that patients may take advantage of online ART in pathologies where standard RT treatments ensure high probability of care. In this perspective, it is necessary to define new metrics able to quantify the quality of each single RT treatment fraction, on the basis of the anatomical variations observed on the daily positioning imaging and their potential impact on dose distribution.

Classifying the quality of a treatment fraction is a very challenging aspect, as at this stage, the clinical impact that can have a non-optimal delivery of a treatment fraction is unknown. However, in the perspective of defining new correlations between the quality of treatment delivery and the clinical outcomes in the future, it is of utmost importance to immediately establish clear criteria to quantify the quality of a treatment fraction delivery.

In this methodological study, we proposed a new metric that quantifies the quality of the right breast treatments based on the value of PTV coverage and the related hot spot: such assumption can be considered reasonable in the right breast, as all the dose constraints related to OARs are widely met using IMRT modality (as reported in **Table 1**) and target coverage remains the only matter of concern.

Extending such metric to a larger cohort of patients with long follow-up could be interesting to investigate if local recurrence would be more present in patients with a higher number of suboptimal fractions.

On the basis of the metric defined in this study, we observed that patients with larger CTV are more prone to experiment target under-coverage due to inter-fraction variability: such type of patients could take advantage of online ART treatments. The study of V95% (PTV) variation has also led to the observation that the value of V95%(PTV)% >95% can be maintained for at least 90% of the treatment fractions if an initial objective goal of V95% (PTV) >98% is reached during the initial planning phase.

Once a metric that classifies the different treatment fractions is identified, it is important to identify predictors based on daily imaging that can alert radiation therapists on the possibility of delivering suboptimal fractions.

To be effective in clinical practice, such indicators should be easy and quick to calculate, to represent a reliable tool also in case of choosing to switch from a conventional treatment to an adaptive one.

In this experience, we observed that the variation of target coverage is correlated with the body variation along the beam axis containing the highest MU: in particular, the probability of observing a PTV under-coverage larger than 2% is equal to 2%, 12%, and 50% in the case of 1 mm, 3 mm, and 5 mm of body variation, respectively.

If confirmed on a larger cohort of patients, such correlation could become a reliable support to the RTTs, allowing the determination of clear thresholds easily identifiable in positioning imaging, beyond which clinician support is required before delivering the treatment fraction.

The main limitation of this study is obviously the reduced number of patients analyzed, mainly related to the recent clinical implementation of this new cutting-edge technology: it is important to remark that the only purpose of this study is to propose new methodological indications on how to approach and manage these novel technologies dedicated to daily ART. The preliminary results here reported require testing on larger cohorts of patients before being considered reliable for clinical use.

By increasing the number of patients enrolled, it will be possible in the near future to elaborate on a predictive model focused on the direct prediction of events considered unacceptable and requiring online adaptation: such a model will be feasible following the same methodology present in this experience once the number of adverse events are statistically sufficient.

Another source of potential uncertainty involves the impact of the breathing motion, which could lead to body variations not due to patient positioning, thus influencing the findings of the study. To limit such aspect, an accurate patient selection was carried out during CT simulation and long CBCT acquisition time was used during treatment, as detailed in the Materials and Methods section.

A last critical point that has to be noted is related to the arbitrary choice of 2% as the limit threshold to consider a fraction as not optimal: also, this value was chosen to propose a new methodology, allowing us to obtain a sufficient number of events in the minority class to make a feasible logistic regression model; a more precise cutoff value can be determined if a larger number of patients is considered. To the best of our knowledge, this represents the first experience that proposes the idea of correlating anatomical variations observed on daily imaging with dose variations in the treatment plan.

## Conclusion

In this methodological study, a novel strategy to identify treatment fractions that may benefit online ART was proposed for patients affected by early right breast cancer. During the RT treatment, the measurement of body difference between daily CBCT and planning CT along the beam axis with the highest MU can be considered as an indirect estimator of V95% PTV variation: a difference larger than 3 mm will result in a V95% decrease of more than 2%. A larger number of observations are recommended before translating the findings of this study in clinical practice.

## Data Availability Statement

The raw data supporting the conclusions of this article will be made available by the authors, without undue reservation.

## Ethics Statement

Ethical review and approval were not required for the study on human participants in accordance with the local legislation and institutional requirements. Written informed consent to participate in this study was provided by the participants’ legal guardian/next of kin.

## Author Contributions

DC, MI, and FC conceptualized the work. GM and VV supervised the work. DaP, FQ, MM, and FT did the data collection. DoP, CV, and SM did the data analysis. DC, MI, AB, AR, AD’A, and CD wrote and revised the manuscript. All authors contributed to the article and approved the submitted version.

## Conflict of Interest

The authors declare that the research was conducted in the absence of any commercial or financial relationships that could be construed as a potential conflict of interest.

## Publisher’s Note

All claims expressed in this article are solely those of the authors and do not necessarily represent those of their affiliated organizations, or those of the publisher, the editors and the reviewers. Any product that may be evaluated in this article, or claim that may be made by its manufacturer, is not guaranteed or endorsed by the publisher.
